# Effect of the Addition of Re on the Microstructure and Phase Composition of Haynes 282: Ab Initio Modelling and Experimental Investigation of Additively Manufactured Specimens

**DOI:** 10.3390/ma16124419

**Published:** 2023-06-15

**Authors:** Antoni Wadowski, Jan S. Wróbel, Milena Koralnik, Ryszard Sitek

**Affiliations:** Faculty of Materials Science and Engineering, Warsaw University of Technology, Wołoska 141, 02-507 Warsaw, Poland

**Keywords:** ab initio calculation, short-range ordering, rhenium effect, nickel alloys, additive manufacturing

## Abstract

Interactions in a multicomponent Ni-Cr-Mo-Al-Re model alloy were determined by ab initio calculations in order to investigate the Re doping effect on Haynes 282 alloys. Simulation results provided an understanding of short-range interactions in the alloy and successfully predicted the formation of a Cr and Re-rich phase. The Haynes 282 + 3 wt% Re alloy was manufactured using the additive manufacturing direct metal laser sintering (DMLS) technique, in which the presence of the (Cr_17_Re_6_)C_6_ carbide was confirmed by an XRD study. The results provide useful information about the interactions between Ni, Cr, Mo, Al, and Re as a function of temperature. The designed five-element model can lead to a better understanding of phenomena that occur during the manufacture or heat treatment of modern, complex, multicomponent Ni-based superalloys.

## 1. Introduction

Ni-based superalloys are widely used in aviation and power generation industries because of their excellent mechanical properties, which are maintained even at high temperatures, and because of their good oxidation resistance and manufacturability. In order to further improve the efficiency of turbines, materials are needed that can withstand extreme temperatures during service under severe stresses and corrosive environments. This can be achieved by optimising the chemical composition of the alloy.

One such superalloy is Haynes 282, for which its chemical composition was designed to achieve a desirable combination of strength, thermal stability, and fabricability [1]. Similarly to other gamma-strengthened Ni-based superalloys, Haynes 282 acquires its mechanical properties by the (i) precipitation of the gamma phase (addition of Al and Ti), (ii) solid solution strengthening (Cr, Co, and Mo), and (iii) the presence of carbides and borides.

By optimising the Al and Ti content of Haynes 282, the volume fraction of the gamma phase is relatively low (19% in a fully aged condition); this is to prevent the phenomenon of strain-age cracking [1] and to achieve good weldability. The impact of a lower volume fraction of gamma is offset by the addition of 8.5 wt% Mo, which plays an important role in maintaining high creep strength via solid solution strengthening, especially near the higher end of the anticipated temperature range [2]. The alloy’s good weldability means that it lends itself to additive manufacturing (AM) by powder bed fusion, which opens up new possibilities for designing parts and material microstructure.

Rhenium as an alloying element increases the creep properties [3,4,5,6,7], fatigue strength, oxidation, and corrosion resistance [4] of Ni-based superalloys. After a debate over the mechanisms underlying the Re effect, it was shown that the improvement in creep strain rate results directly from Re enrichment to partial dislocations, hindering their movement [8]. Besides its direct interactions with dislocations, Re significantly enhances the stability of gamma precipitates and suppresses variations in composition within the gamma matrix [9]. Re atoms, which have the lowest diffusion coefficient in Ni-based superalloys, slow down diffusion-controlled processes in the microstructure, therefore increasing its stability during exposure to high temperatures [4,9]. It has been shown that Re atoms enrich gamma dendrites in multicomponent Ni alloys [7,10,11,12,13,14], leading to a non-homogeneous distribution of elements between dendritic and interdendritic regions. Moreover, Re leads to the formation of TCP phases, which are harmful to high-temperature properties [4,5,11,15,16]. Although Re’s tendency toward microsegregation [7,10,11,12,13,14] and crystal lattice occupying sites [17] is well established, a quantitative analysis of Re’s interactions in multicomponent Ni-based superalloys requires a more thorough examination.

Other works that investigate Re’s effect on the phase composition of Ni alloys often focus on thermodynamic calculations based on the CALPHAD method (e.g., Refs. [14,18]), which cannot provide direct information about interactions between specific atoms. Moreover, the ab initio models applied in previous articles are usually limited to a temperature of 0 K and often narrowed to a single-phase investigation, for example, the study of the spatial arrangement of atoms restricted to the L1_2_ Ni_3_Al phase in Ref. [17]. In addition, although attempts were carried out to determine interactions between elements in pure Haynes 282 [19], the results are limited to a temperature of 0 K. Ab initio based Monte Carlo simulations are proven to accurately describe temperature-dependent interactions between elements in multicomponent systems [20,21]. This knowledge is necessary to further enhance the properties of Ni-based superalloys by optimising their chemical composition.

The aim of this study was to improve our knowledge of the interactions between atoms and phase stability in rhenium-doped additively manufactured Haynes 282 alloy. To investigate the rhenium effect on the alloy, a theoretical Ni-Cr-Mo-Al-Re model was developed using a combination of density functional theory, cluster expansion, and Monte Carlo methods. To the authors’ knowledge, such a system and the effect of Re on Haynes 282 have never been previously investigated. Simulated interactions between elements were compared with experimentally observed phases in the produced Haynes 282 + 3 wt% Re alloy.

## 2. Materials and Methods

To investigate the effect of adding Re to Haynes 282 alloy (chemical composition shown in Table 1), the cluster expansion model for the face-centred cubic (fcc) Ni-Cr-Mo-Al-Re system was developed, in which effective cluster interactions were determined based on the density functional theory (DFT).

Simulations for systems with different chemical compositions (Table 2) were carried out. To reflect Haynes 282 properties using a five-element cluster expansion model, the most important alloy elements were chosen: Ni, Cr, Mo, and Al. Chromium and molybdenum are amongst the most abundant elements in the Haynes 282, and their fraction corresponded to the reference alloy’s composition [1]. It is worth noting that there is a higher percentage of Co than Mo in Haynes 282 (10 and 8.5 wt%, respectively). However, molybdenum was chosen to be included in the model. It plays a vital role in defining the creep properties of Haynes 282 [1] while also influencing the phase stability of Ni-based superalloys, i.e., leading to μ phase formation [22]. Because both Al and Ti mainly enrich the gamma L1_2_-Ni_3_(Al, Ti) phase in Ni-based superalloys [23] and occupy similar positions in its crystal structure [17], the concentration of Al was set as a sum of Al and Ti fractions in the Haynes 282 alloy. Other elements present in the reference alloy (e.g., Co, Fe, Mn) were entirely replaced by Ni. In subsequent variations (Table 2), Re was added at the expense of the at.% of Ni, which imitated the experimental Re doping of Haynes 282 powder.

DFT calculations were performed using the Vienna Ab initio Simulation Package (VASP) version 5.4.4 [24,25], with cutoff energy equal to 400 eV, and the Monkhorst–Pack mesh [26] of *k* points in the Brillouin zone, with a *k*-mesh spacing of 0.2 ${Å}^{-1}$, which corresponds to 12 × 12 × 12 *k*-point meshes for a four-atom fcc cubic cell. The total energies were converged to 10^−5^ eV, and the force components in calculations involving full cell relaxation were relaxed to 10^−3^ eV/Å. Alloy Theoretic Automated Toolkit (ATAT) version 3.04 [27] was used to develop the cluster expansion (CE) model for the fcc Ni-Cr-Mo-Al-Re system based on DFT calculations for 403 binary, 771 ternary, and 494 quaternary structures. In CE formalism, the enthalpy of the mixing of an alloy described by a vector of the configurational variables $\vec{\sigma }$ was expressed as [27,28,29]

(1) $$H_{mix}\left(\vec{\sigma }\right)=\underset{\omega }{\sum }m_{\omega }J_{\omega }\langle \Gamma_{\omega ’}\left(\vec{\sigma }\right)\rangle$$

where summation is performed over all clusters, *ω*, with multiplicity *m_ω_. J_ω_* denotes concentration-independent effective cluster interaction parameters (ECIs), and $\langle \Gamma_{\omega ’}\left(\vec{\sigma }\right)\rangle$ denotes cluster functions defined as the products of the point functions of occupation variables on a specific cluster ω averaged over all clusters ω’ that are equivalent to cluster ω by symmetry. In the CE model for the fcc Ni-Cr-Mo-Al-Re system, we used 50 two-body, 100 three-body and 35 four-body ECIs. The cross-validation score between the enthalpies of mixing that are computed using DFT and CE was 22.3 meV. To investigate the phase stability of fcc Ni-Cr-Mo-Al-Re alloys, the CE model was next applied in Monte Carlo (MC) simulations, which were conducted using the ATAT code [27] by quenching the system from a temperature of 2000 K to 200 K with a temperature step equal to 100. Simulation cells were 20 × 20 × 20 fcc unit cells containing 8000 atoms. The correlation functions and enthalpies of mixing were calculated by averaging the MC results over 2000 MC steps per atom at each temperature.

Chemical ordering in alloys was investigated by the calculation of Warren–Cowley short-range order (SRO) parameters, which are defined as

(2) $$\alpha_{n}^{ij}=1-\frac{y_{n}^{ij}}{c_{i}c_{j}}$$

where i and j are the n-th nearest neighbour atoms, $c_{i}$ and $c_{j}$ are the concentrations of atoms i and j, respectively, and $y_{n}^{ij}$ is the average pair probability, which can be obtained from the average point and pair correlation function as in Refs. [29,30,31].

Samples of the modified alloy were manufactured using the DMLS technique. To obtain a powder of Haynes 282 + 3 wt% Re, a pure alloy powder manufactured by Höganäs AB (Höganäs, Sweden) was mixed with 99,99% Re powder (KAMB Import-Eksport, Warsaw, Poland) in a mass proportion of 50/50 in a Fritsch Pulverisette 5 planetary ball mill (Weimar, Germany), and the parameters are shown in Table 3. After this step, additional pure Haynes 282 powder was added to achieve a mixture of Haynes 282 with 3 wt% Re.

The 10 × 20 × 15 mm Haynes 282 + 3 wt% Re samples were manufactured using an EOS M100 3D printer (EOS GmbH, Krailling, Germany). The additive manufacturing of the samples was carried out using a laser power of 90 W, scanning speed of 1200 mm/s, and powder layer thickness equal to 20 μm. This operation was followed by a standard heat treatment for Haynes 282. Firstly, solution annealing at 1149 °C for 2 h followed by water quenching was carried out. Then, samples were subjected to a two-stage age-hardening treatment: 1010 °C for 2 h and air-cooled and 788 °C for 8 h and air-cooled.

Phase analysis of the investigated material was performed by means of X-ray diffraction (XRD) at room temperature using a Bruker D8 Advance diffractometer (Billerica, MA, US) with filtered CuKα radiation (λ = 0.154056 nm). The results were recorded by stepwise scanning in a 2Θ range of 20°÷120°, with a step size of 0.05°, a count time of 3 s per step, and a voltage of 40 kV. The XRD patterns were analysed using Bruker EVA V3.0 software and a PDF-2 database.

## 3. Results and Discussion

### 3.1. Ab-Initio Modelling

**Figure 1:**
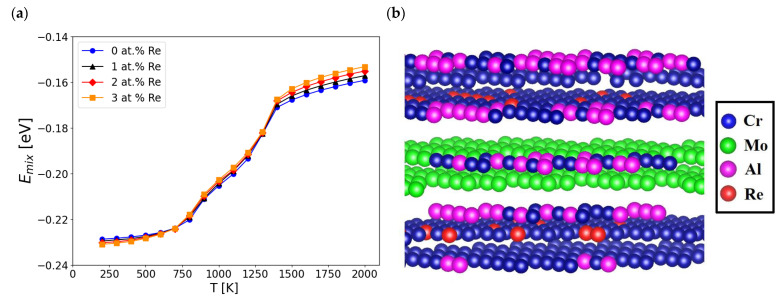
(**a**) Enthalpies of mixing (Emix) for alloys with chemical compositions listed in Table 2 and (**b**) Ni66Cr22Mo5Al6Re1 structure visualisation (which correspond to Haynes 282 + 3 wt% Re, listed in Table 2) at a temperature of 300 K. Ni atoms were excluded from the visualisation for clarity.

As shown in Figure 1a, the addition of Re to Haynes 282 practically did not change the alloy’s enthalpy of mixing for temperatures below 1300 K. In higher temperatures, the enthalpy of mixing increases with the Re concentration in the alloy. An order–disorder transformation also occurred at the same temperature (approximately 1350 K).

In Figure 1b, a representative structure of the alloy containing 1 at. % Re generated using MC simulations at 300 K is shown. There are visible regions with the coexistence of Cr and Re atoms as well as the coexistence of Al and Cr atoms.

**Figure 2:**
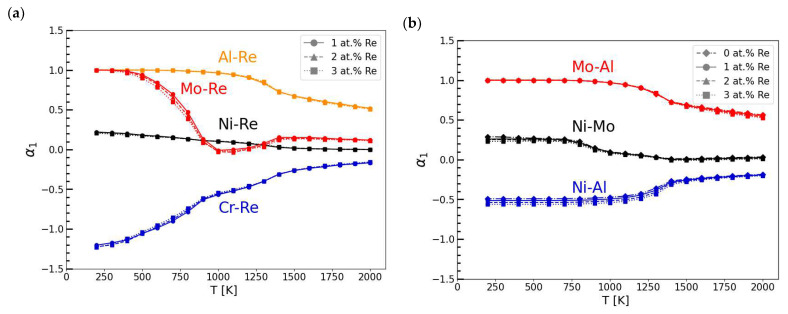

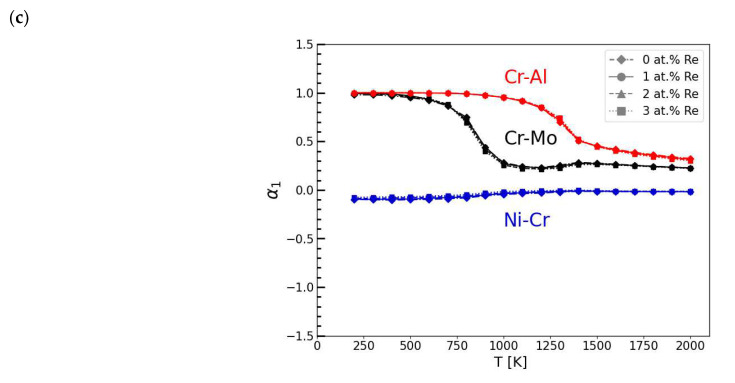
Calculated short-range order (SRO) parameters for pairs of atoms in the 1st nearest neighbour coordination shell (*α_1_*): (**a**) Re and X atoms, where X = {Ni, Cr, Mo, Al}; (**b**) Ni-Mo, Ni-Al, and Mo-Al; and (**c**) Cr-Al, Cr-Mo, and Ni-Cr pairs.

**Figure 3:**
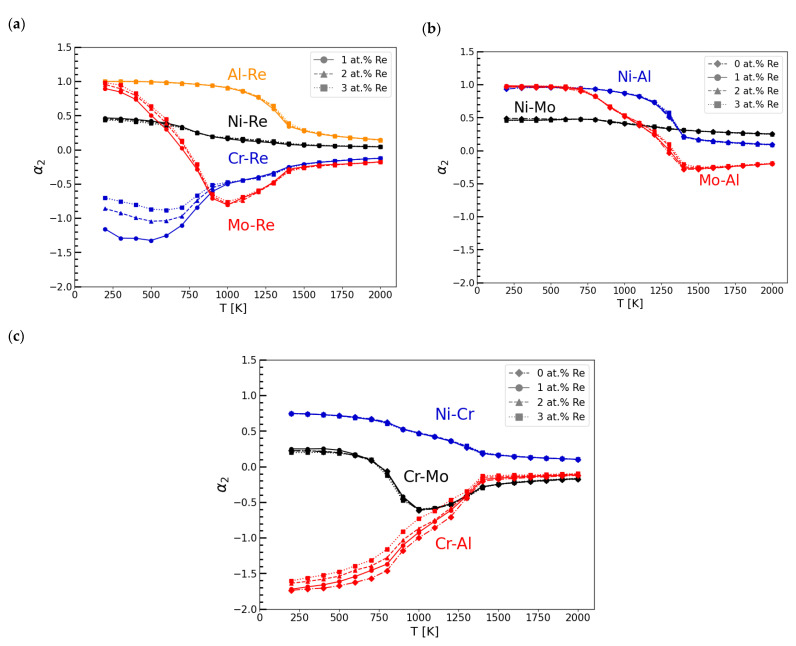
Calculated SRO parameters for pairs of atoms in the 2nd nearest neighbour coordination shell (*α_2_*): (**a**) Re and X atoms, where X = {Ni, Cr, Mo, Al}; (**b**) Ni-Mo, Ni-Al, and Mo-Al; and (**c**) Cr-Al, Cr-Mo, and Ni-Cr pairs.

To study the atomic ordering in Ni-Cr-Mo-Al-Re alloys as a function of Re concentration and temperature, the SRO parameters were calculated using Equation (2) based on average correlation functions from MC simulations. The values of SRO for the pairs of atoms in the first and second nearest neighbour coordination shell are shown in Figure 2 and Figure 3, respectively.

The lower and more negative the SRO value, the stronger the attractive forces between pairs of atoms, and vice versa. Therefore, systems with small values of SRO parameters interactions value are more likely to create disordered, or when the SRO is negative and significantly lower, even ordered solid solutions, leading to stable phase formation. An increasing and positive SRO value means stronger repulsive forces between pairs of atoms, leading to the segregation of the mixture.

As presented in Figure 2a, the values of SRO parameters for the first nearest neighbour coordination shell (*α_1_*) are strongly negative for the Cr-Re system throughout the investigated temperature range. The forces between the Cr and Re atoms can cause the formation of stable and ordered structures, such as intermetallic phases. The effect of attractive forces between Cr and Re atoms can be observed in Figure 1b, in which Re is distributed in the direct neighbourhood of Cr atoms.

The interactions of other pairs of atoms (Al-Re, Mo-Re, and Ni-Re) have positive values of *α_1_* throughout the temperature range, except for the Mo-Re pair. At temperatures near 1100 K, the *α_1_* values for the Mo and Re atoms have slightly negative values. This suggests that during alloy exposure at that temperature (i.e., during casting or heat treatment), it is possible for phases rich in Mo and Re to precipitate. The *α_1_* characteristic of the Ni-Re pair indicates that because of slightly positive values lower than 0.25, Ni and Re create a disordered solid solution in which the elements show little tendency to segregate.

Based on the modelling results, it is suspected that Al and Re would strongly segregate from one another. Therefore, Re is not supposed to be present within an ordered gamma L1_2_ Ni_3_Al structure (Figure 1b), which agrees with experimental investigations of other Ni-based superalloys [9]. As shown in Figure 2b, the *α_1_* values for the Ni-Al system were the lowest of the investigated atomic Al pairs, making this element the one most likely to exist in phases with Ni. This observation is common and remains the basic principle behind nickel superalloys’ precipitation strengthening.

Furthermore, because of high values of *α_1_* in systems Mo-Al (Figure 2b) and Cr-Al (Figure 2c), it can be concluded that Mo and Cr would not occur in the first nearest neighbour positions of Al.

As presented in Figure 3a, the values of SRO parameters for the second nearest neighbour coordination shell (*α_2_*) for Re and other elements present in the model (Ni, Cr, Mo, and Al) show a similar tendency to the results for the 1st shell (Figure 2a). However, the second shell SRO parameter for Mo-Re has more negative values within a broader temperature range. The *α_2_* for Ni-Al presented in Figure 3b has positive values. This suggests that Ni and Al atoms will not likely be present in their second neighbourhood positions. This observation is in good agreement with the structure of the ordered gamma L1_2_ Ni_3_Al phase present in Haynes 282. It can be observed that in the case of the Cr-Al pair, the SRO parameter for the second nearest neighbour coordination shell (Figure 3c) has negative values, contrary to the results for the first shell (Figure 2c). In effect, some Cr can be present near Al atoms (Figure 1b), preferably in their second nearest neighbour position. The addition of Re reduces this tendency, leading to a less negative *α_2_* for the Cr-Al system.

### 3.2. XRD Results and Comparison with MC Simulations

**Figure 4:**
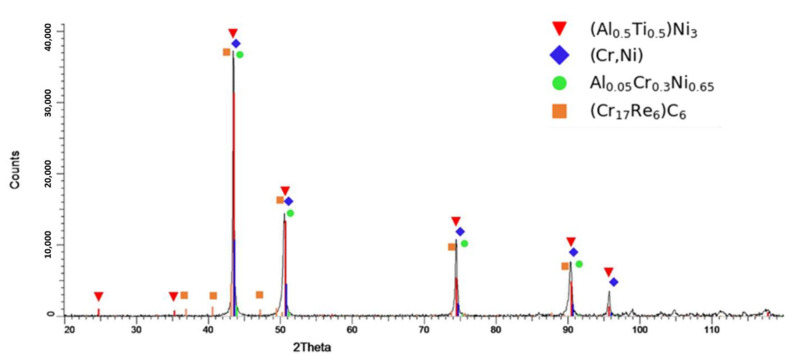
X-ray diffractogram pattern of Haynes 282 + 3 wt% Re.

Figure 4 shows a diffraction pattern of the manufactured Haynes 282 + 3 wt% Re that is examined along the *Z*-axis of a printed sample with the pattern of the Ni_3_(Al_0.5_Ti_0.5_) phase in the matrix solution of Ni and Cr. A broadening of the major diffraction peaks was observed, probably due to the presence of additional phases. Based on the positions of the diffraction peaks and the known chemical composition of the tested material and using the PDF-2 database, the Al_0.05_Cr_0.3_Ni_0.65_ and C_6_(Cr_17_Re_6_) phases were characterized by the highest degree of adjustment. The identified phases show a cubic crystal system with lattice parameters of 3.566 Å and 10.890 Å, respectively. The performed tests did not reveal the presence of Mo-containing phases.

The existence of Cr- and Re-rich C_6_(Cr_17_Re_6_) phases in the considered Ni-based alloy agrees with the Monte Carlo results showing a strong attraction of Cr and Re atoms (Figure 2a and Figure 3a). It should be noted that the theoretical investigation of the alloy’s stability in the presence of carbon was out of the scope of this work. However, the Monte Carlo simulations show that Cr- and Re-rich phases should be able to form even without C. It is worth noting that strong attraction forces between Cr and C are experimentally proven by the existence of Cr-rich carbides in Ni-based superalloys [32,33].

Moreover, the presence of the Al_0.05_Cr_0.3_Ni_0.65_ phase further confirms the Monte Carlo results obtained for the investigated alloy. Unveiled significant attractive forces between Cr and Al atoms in the second nearest neighbour coordination shell (Figure 3c) can explain the formation of a stable phase that is rich in those elements.

## 4. Conclusions

To conclude, the fcc Ni-Cr-Mo-Al-Re model was developed to investigate interactions between atoms in the Re-modified Haynes 282 alloy. The addition of Re as an alloying element to Haynes 282 preserves the character of the atomic interactions between the most important elements in the alloy, such as Ni, Cr, Mo, and Al. Therefore, Haynes 282 can be modified using Re to achieve desirable properties that are maintained during heat treatment procedures, similarly to those of an unmodified alloy. The theoretical multicomponent model of the Re-doped alloy unveiled significant attractive forces between Re and Cr atoms, which indicate the tendency of these elements to form stable phases. This is in agreement with the presence of the (Cr_17_Re_6_)C_6_ carbide, which was detected in the XRD study of the additively manufactured Haynes 282 + 3 wt% Re alloy. Moreover, the negative values of SRO parameters for the Al-Cr pair in the second nearest neighbour coordination shell explained the presence of the Al_0.05_Cr_0.3_Ni_0.65_ phase in the X-ray diffractogram pattern of the material.

## Figures and Tables

**Table 1:** Chemical composition of Haynes 282 alloy in weight and atomic percentage. The concentration of Ni is set as the balance, taking the maximum concentration values of Fe, Mn, Si, and C.

	Cr	Co	Mo	Ti	Al	Fe	Mn	Si	C	B
wt% [1]	20	10	8.5	2.1	1.5	1.5 *	0.3 *	0.15 *	0.06 *	0.005
at.%	22.1	9.8	5.1	2.5	3.2	1.5 *	0.3 *	0.3 *	0.3 *	0.03

* Maximum.

**Table 2:** Chemical compositions of investigated Ni-Cr-Al-Re alloy models.

wt% of Re	0	3	6	9
Ni (at.%)	67	66	65	64
Cr (at.%)	22	22	22	22
Mo (at.%)	5	5	5	5
Al (at.%)	6	6	6	6
Re (at.%)	0	1	2	3

**Table 3:** Ball milling parameters used for mixing Haynes 282 and Re powder.

Parameter	Description
Grinding cycles	Milling: 200 rpm, 10 min Pause time: 20 min
Total grinding time	4 h
Balls	Material: tungsten carbide Diameter: 3 mm
Ball-to-powder mass ratio (BPR)	5:1

## Data Availability

Data is contained within this article.
